# Bladder chondrosarcoma plus urothelial carcinoma in recurred transitional cell carcinoma of the upper urinary tract: a case report and literature review

**DOI:** 10.1186/s12957-016-1021-3

**Published:** 2016-10-20

**Authors:** Min Hyun Cho, Sung Han Kim, Weon Seo Park, Jae Young Joung, Ho Kyung Seo, Jinsoo Chung, Kang Hyun Lee

**Affiliations:** 1 Department of Urology, Seoul National University Hospital, Seoul, South Korea; 2 Department of Urology, Center for Prostate Cancer, Research Institute and Hospital of National Cancer Center, 323 Ilsan-ro, Ilsandong-gu, Goyang-si, Gyeonggi-do 410-769 South Korea; 3 Department of Pathology, Center for Prostate Cancer, National Cancer Center, Goyang, South Korea

**Keywords:** Urothelial carcinoma, Chondrosarcoma, Bladder, Ureter, Recurrence

## Abstract

**Background:**

Sarcomatoid urothelial carcinoma (SUC) is a rare malignant neoplasm of the urinary bladder comprising 0.2–0.6 % of all histological bladder tumor subtypes. It presents as a high-stage malignancy and exhibits aggressive biological behavior, regardless of the treatment employed. It is defined as histologically indistinguishable from sarcoma and as a high-grade biphasic neoplasm with malignant epithelial and mesenchymal components. The mean age of patients presenting with SUC is 66 years, and the male-to-female ratio is 3:1. In addition, gross hematuria is usually present. The prognosis of SUC is poorer than that of typical urothelial carcinoma because of uncertainty concerning the optimal treatment regimen.

**Case presentation:**

We report the case of a 77-year-old woman with SUC containing a chondrosarcoma component who, 12 years previously, had undergone a nephroureterectomy for pT3N0M0 ureter cancer of the contralateral upper urinary tract. From the 4th year of follow-up after nephroureterectomy, multiple recurrent bladder tumors staged as Ta transitional cell carcinoma developed, and six transurethral resections of the bladder (TURB) with multiple intravesical instillations were performed without any evidence of metastases and upper tract recurrences. In 2015, a right partial distal ureterectomy and an additional TURB were performed due to a papillary mass at the right contralateral ureterovesical junction of the bladder, which was confirmed as a high-grade pT1 transitional cell carcinoma. After a further 2 years of follow-up, total pelvic exenteration with an ileal conduit diversion was performed to remove the mass, which was a pT4N0M0 tumor composed of carcinomatous and sarcomatous elements compatible with a sarcomatoid carcinoma including grade 3 transitional cell carcinoma and chondrosarcoma. Immunohistochemical examination showed that tumor cells were positive for vimentin and p63 and negative for NSE and Cd56 markers. In the first postoperative month, a metastatic lung nodule was detected on chest CT. The patient was scheduled for adjuvant gemcitabine-cisplatin chemotherapy.

**Conclusions:**

The present case was interesting because we cannot be sure if the SUC chondrosarcoma originated from the 12-year-ago proximal ureter tumor, the 2-year-ago contralateral distal ureter tumor, or a new primary bladder tumor. Genetic profiling might have been useful to determine the origin of the SUC chondrosarcoma.

## Background

Sarcomatoid urothelial carcinoma (SUC) is a rare malignant neoplasm of the urinary bladder comprising 0.2–0.6 % of all histological bladder tumor subtypes [1, 2]. It presents as a high-stage malignancy and exhibits aggressive biological behavior, regardless of the treatment employed. It is defined as histologically indistinguishable from sarcoma and as a high-grade biphasic neoplasm with malignant epithelial and mesenchymal components. In the past, when SUC showed specific mesenchymal differentiation such as that in chondrosarcoma, some pathologists have preferred to use the term “carcinosarcoma” [3]. The mean age of patients presenting with SUC is 66 years, and the male-to-female ratio is 3:1. In addition, gross hematuria is usually present. The prognosis of SUC is poorer than that of typical urothelial carcinoma because of uncertainty concerning the optimal treatment regimen.

Here, we report the case of a 77-year-old woman with SUC containing a chondrosarcoma component who, 12 years previously, had undergone a nephroureterectomy for pT3N0M0 transitional cell carcinoma of ureter cancer of the contralateral upper urinary tract.

## Case presentation

**Fig. 1:**
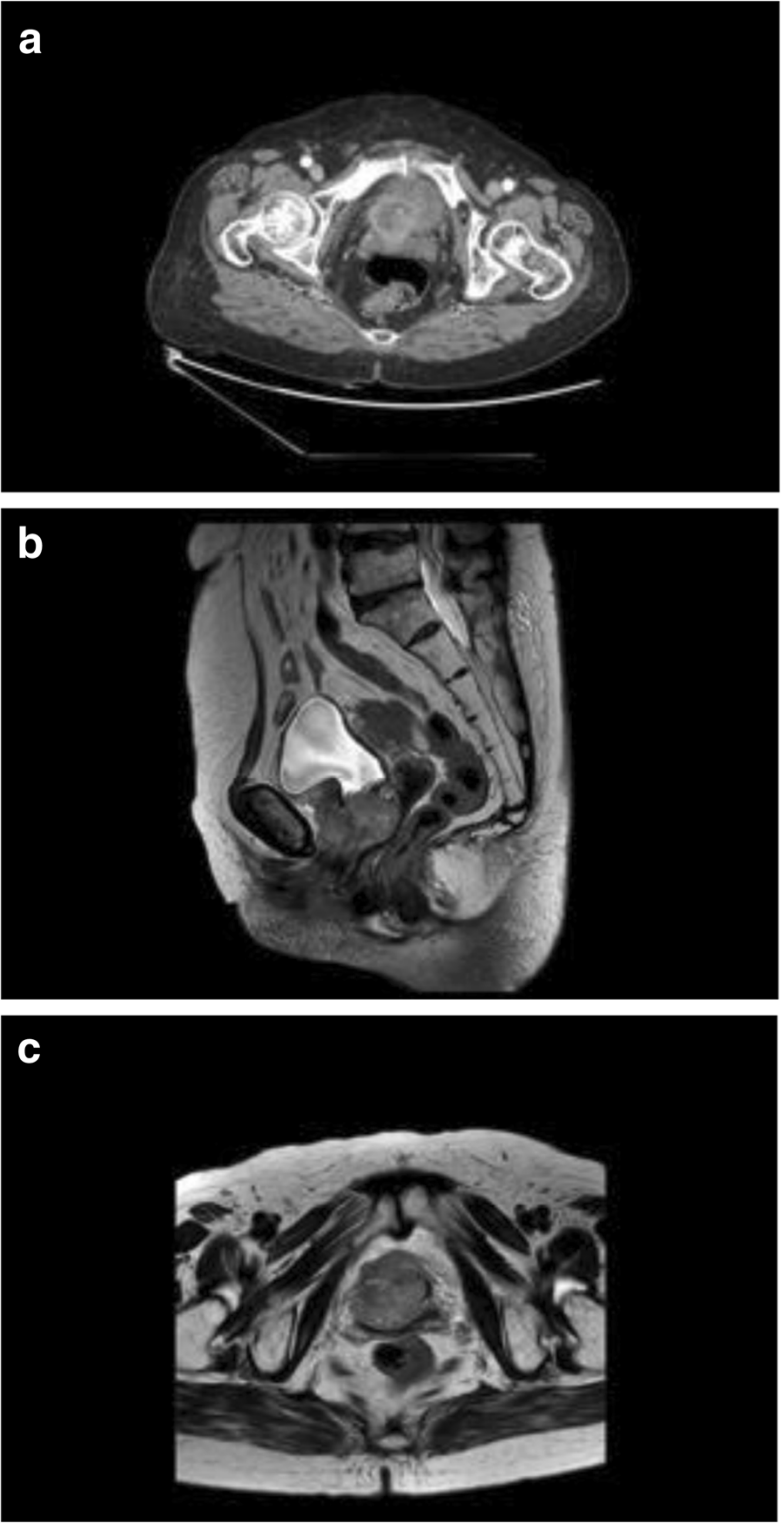
Preoperative gynecologic MRI and abdominal CT images. **a** A 4-cm recurred tumor involving the bladder neck, urethra, and upper vagina (horizontal view, abdominal pelvis CT). **b** An increased recurred tumor involving the bladder neck, urethra, and upper vagina (4.6 cm). There were no newly appearing lesions in the pelvic cavity indicating a lack of metastasis (sagittal view, MRI gynecology). **c.** A 4-cm recurred tumor involving the bladder neck, urethra, and upper vagina. There were no other newly appearing lesions in abdominopelvic cavity indicating a lack of metastasis (horizontal view, MRI gynecology)

**Fig. 2:**
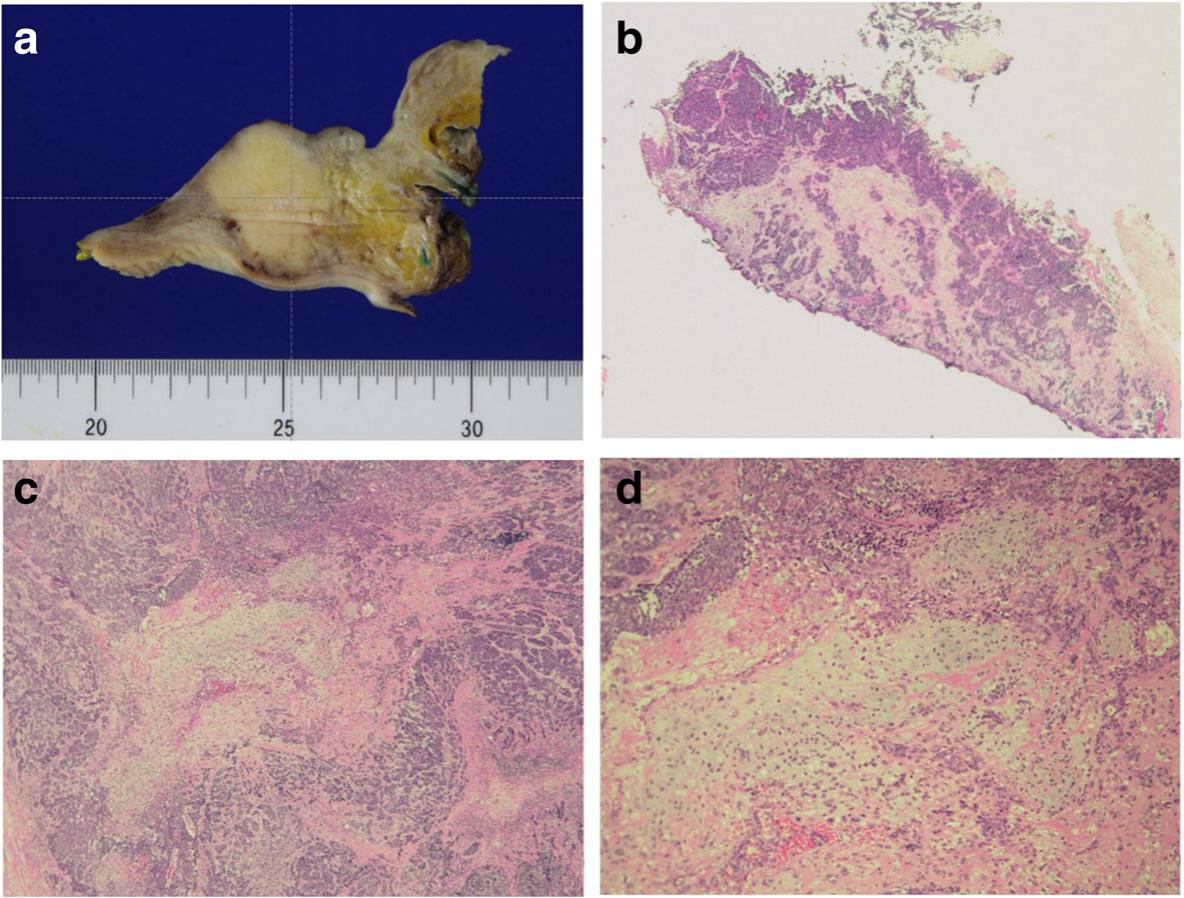
Representative photomicrographs of the urothelial carcinoma in the bladder. **a** Sectional gross photograph of the specimen. The *upper part* shows the bladder and the *lower part* shows the vagina. There is a well-demarcated round mass under the bladder mucosa. **b** Photomicrograph of the TURB (×40). A poorly differentiated urothelial carcinoma is noted on the mucosa and in the subepithelial connective tissue. **c** A poorly differentiated urothelial carcinoma surrounded by a sarcomatoid component (*center*, chondrosarcoma; ×40). **d** High-powered view of the chondrosarcoma component (hematoxylin and eosin staining; ×100)

A 77-year-old woman visited our hospital for multiple recurred bladder tumors. She had a history of well-controlled hypertension. Twelve years previously, at a different hospital, she underwent a left nephroureterectomy with adjuvant chemotherapy (6 cycles of monthly Gemzar-cisplatin combination chemotherapy and 3 cycles of monthly Gemzar-carboplatin chemotherapy) for pT3N0M0 poorly differentiated UC of the ureter. No further recurrence was detected during the routine follow-ups. On the 4th year of follow-up after nephroureterectomy, she developed gross hematuria and left lower quadrant discomfort that had persisted for 2 months. She was referred to our institution after cystoscopy revealed multiple recurred papillary bladder tumors. Transurethral resection of the bladder tumors (TURB) was performed followed by 6 cycles of intravesical instillation therapy. Histologically, the tumor was a high-grade pTa transitional cell carcinoma (TCC). During the next 6 years, a further five TURB operations were performed for recurred bladder tumors, and three different courses of postoperative intravesical instillations using mitomycin-C, epirubicin, or BCG agents were performed after each TURB. Pathologically, the tumors were low- to high-grade pTa TCC with or without carcinoma in situ. In 2015, a gross hematuria developed, and routine cystoscopy follow-up revealed a papillary mass appearing at the right contralateral ureterovesical junction of the bladder. Computed tomography (CT) did not reveal any metastases or lymph node enlargements. She underwent a right, 1.5-cm length, partial, distal ureterectomy and an end-to-end ureteroureterostomy with an additional TURB at the right ureteral orifice. Frozen section analysis and final pathology confirmed that the distal and proximal ureteral margins and the resected bladder sections were negative for tumor cells. The mass was confirmed as a high-grade pT1 TCC. After a further 2 years of follow-up, CT revealed a 4-cm, submucosal, invasive, non-pedunculated, elevated bladder mass with an intact mucosa involving the bladder neck, urethra, and upper vagina (Fig. 1). Total pelvic exenteration with an ileal conduit diversion was performed to remove the mass, which was a pT4N0M0 tumor composed of carcinomatous and sarcomatous elements compatible with a sarcomatoid carcinoma including grade 3 TCC and chondrosarcoma (Fig. 2). Immunohistochemical examination showed that tumor cells were positive for vimentin and p63 and negative for NSE and Cd56 markers. In the first postoperative month, a metastatic lung nodule was detected on chest CT. The patient underwent two more cycles of adjuvant Gemzar-cisplatin chemotherapy, but further chemotherapy was not performed because of her poor general condition and neutropenic side effects.

### Discussion

SUC is an unusual malignancy containing both carcinomatous and sarcomatous components. It is a rare, but aggressive, bladder cancer, comprising <1 % of all bladder cancers [2]. In most reported SUC cases, the epithelial component was UC, although squamous cell and small-cell carcinoma components have been reported. The mesenchymal component varies from homogeneous sarcoma to heterotopic elements, such as malignant bone, cartilage, and other mesenchymal tissues. Radiation and intravesical cyclophosphamide instillation therapy are typical risk factors. In addition, a recent multicenter study showed that 50–79 % of patients with SUC were current smokers [2]. Nodal and visceral metastases are often present at diagnosis (>20 % of patients). However, the present case did not present with any of these risk factors.

A multimodal approach that includes definitive surgery, local radiation therapy, and chemotherapy is often used for locally advanced disease. For patients with sarcomatoid carcinoma of the bladder, previous studies and reviews have indicated that a GC-based regimen is well tolerated and effective, given its ability to induce complete remission [2–5]. Chemotherapy, therefore, has been the mainstay treatment for metastatic disease; however, the response rates have been varied. The 5-year cancer-specific survival after cystectomy is 20 %, and the median overall survival is 14 months. SUC tends to present at an advanced stage and is associated with a poor prognosis.

According to the WHO classification [4], macroscopically, sarcomatoid carcinomas are large and polypoid with infiltrative margins, a fleshy appearance, and the presence of hemorrhage, necrosis, and cavitation. Microscopically, conventional urothelial, squamous, glandular, or small-cell components may be mixed with sarcomatous components that usually predominate. The sarcomatoid component consists of high-grade spindle-shaped or pleomorphic cells. Heterologous components can include osteocarcinoma, chondrosarcoma, rhabdomyosarcoma, leiomyosarcoma, liposarcoma, and angiosarcoma [4]. Previous studies have suggested that patients whose tumors harbor heterologous elements may have a worse prognosis compared to those without them. In 80 % of cases, epithelial cells are vimentin positive, which is congruent with the findings in the present case. Conversely, the sarcomatoid component can retain focal expression of high molecular weight cytokeratins, p63, and GATA3.

The histogenesis of SUC is unclear, and Armstrong et al. [5] examined TP53 mutation status and p53 protein expression in both the sarcomatoid and epithelial components of SUC. They suggested a common clonal origin of the phenotypically different tumor components, and that TP53 mutations probably occurred early in the common tumorigenesis of these morphologically distinct tumor components. Subsequently, these lesions were likely exposed to additional stimuli, giving rise to the biphasic phenotype [5].

The present case was interesting because the patient had a history of both high-stage pT3 ureter cancer and contralateral distal ureter tumor. After several endoscopic resections for superficial low-grade recurrent bladder tumors and intravesical instillation therapies with multiple agents, SUC chondrosarcoma occurred in the bladder after cystectomy. We cannot be sure if the SUC chondrosarcoma originated from the proximal ureter tumor that occurred 12 years earlier, the contralateral distal ureter tumor that occurred 2 years earlier, or a new primary bladder tumor. Genetic profiling might have been useful to determine the origin of the SUC chondrosarcoma. However, the patient was diagnosed with metastatic lung cancer during the follow-up and was scheduled to undergo chemotherapy.

## Conclusions

We reported the case of a 77-year-old woman with SUC containing a chondrosarcoma component. Further accumulated cases studies with genetic profiling would improve the understanding of SUC and enable optimal treatment planning.

## Abbreviations

CT: Computed tomography
SUC: Sarcomatoid urothelial carcinoma
TCC: Transitional cell carcinoma
TURB: Transurethral resection of bladder
